# Action and Reaction of Pre-Primary and Primary School-Age Children to Restrictions during COVID-19 Pandemic in Greece

**DOI:** 10.3390/jpm11060451

**Published:** 2021-05-23

**Authors:** Dimitra I. Siachpazidou, Ourania S. Kotsiou, Grigorios Chatziparasidis, Dimitrios Papagiannis, George D. Vavougios, Eudoxia Gogou, Vasileios T. Stavrou, Konstantinos I. Gourgoulianis

**Affiliations:** 1 Department of Respiratory Medicine, Faculty of Medicine, University of Thessaly, Biopolis, 41110 Larissa, Greece; sidimi@windowslive.com (D.I.S.); dantevavougios@hotmail.com (G.D.V.); vasileiosstavrou@hotmail.com (V.T.S.); kgourg@med.uth.gr (K.I.G.); 2 Faculty of Nursing, School of Health Sciences, University of Thessaly, Gaiopolis, 41110 Larissa, Greece; 3 Department of Primary Ciliary Dyskinesia, School of Medicine, University of Thessaly, 41110 Larissa, Greece; gchatziparasidis@gmail.com; 4 Public Health & Vaccines Lab, Department of Nursing, School of Health Sciences, University of Thessaly, Gaiopolis, 41110 Larissa, Greece; dpapajohn@uth.gr; 5 Department of Physiology, Faculty of Medicine, University of Thessaly, Biopolis, 41500 Larissa, Greece; egogou@uth.gr

**Keywords:** children, coronavirus disease 2019, lockdown, school closure

## Abstract

The fast-spreading coronavirus disease 2019 (COVID-19) pandemic forced countries to apply restrictive measures to counteract it. School closure was quickly adopted by health authorities. We aimed to investigate the compliance of children aged 4 to 12 years with the COVID-19 lockdown restrictions and evaluate the impact of school closure on the children’s educational, social, economic, and psychological outcomes. An online survey was distributed through a social networking platform to parents of pre-primary and primary school-age children. The study period was defined as from 27 November 2020 to 3 December 2020, two weeks after the school closure due to the general lockdown in Greece. This study showed that the school units were well-informed and complied with the protection measures against COVID-19. The pupils quickly adopted the protection measures, even those whose parents suggested masks were less effective. The quarantine-forced school closure highly impacted primary school children’s physical activity, quality of sleep, psychological status, eating habits, academic performance, and household income. Web use showed an increase, with the children over-spending extracurricular time in web activities. Our study highlights the need for long term monitoring of these aforementioned indices, and the development of COVID-19 mitigation measures that carefully incorporate effectiveness and societal impact.

## 1. Introduction

The 2019 coronavirus disease (COVID-19) pandemic forced countries worldwide to apply restrictive measures to avoid the rapid spread of severe acute respiratory syndrome coronavirus-2 (SARS-CoV-2). School closure was considered a proactive measure against the pandemic and was quickly adopted by many governments [1]. These policies were founded on previous experience with mass influenza A epidemics, and aimed at a flat reduction of transmissibility [2,3,4]. While school closure has been adopted by several countries, multiple meta-analyses have indicated a minimal effect on COVID-19’s transmission dynamics and outcomes [5,6,7].

Undoubtedly the school environment helps with learning and positively impacts personality traits and sense of identity, especially in children aged 2 to 10 years old [8]. The UN Educational, Scientific and Cultural Organization estimates that school closure affects the education of 80% of children worldwide [9]. Electronic learning (e-learning) reduces children’s academic performance, especially in those of the first school grades where hand-eye coordination is required for writing [10,11]. Beyond the educational parameters, the ongoing pandemic has led to a new economic recession and economic downturn, increasing the number of children living in poverty [12].

Lockdowns and school closure are expected to have negative psychological, educational, and social consequences for children and their families [1]. The consequent isolation and social limitations would furthermore serve to introduce and enhance various psychological stressors and neuropsychiatric manifestations [13]. It has been reported that due to the pandemic restrictions, children have reduced physical activity as they cannot participate in school or extracurricular activities [14,15,16]. Additionally, isolation under stressful conditions could have an impact on sleep quality [17]. Parents also report that children manifest increased stress levels and adopt poor eating habits during the quarantine. Stress leads to excessive consumption of foods rich in sugar [18,19] and other simple carbohydrates [20].

There are few comprehensive impact studies on the consequences of school closures among children and parents. The aim of our study was to investigate the compliance of pre-primary and primary school-age children aged from 4 to 12 years with COVID-19 lockdown restrictions and evaluate the short-term impact of school closure on children’s educational, social, economic, and psychological outcomes.

## 2. Materials and Methods

### 2.1. Study Design

An online survey was conducted via a cloud-based short questionnaire on Google Forms, which was disseminated via institutional social accounts on the social networking platform Facebook and focused on the parents of children aged from 4 to 12 years (kindergarten and primary school) without any promise of reward.

The study was conducted two weeks after the school closure by national lockdown from 27 November 2020 to 3 December 2020, to detect short-term effects of school closure due to lockdown on children’s outcomes. Numerous quantitative studies have been conducted worldwide adopting this technique, making it popular and proving its effectiveness on speed and range, convenience, cost, flexibility, and automation [21,22].

We employed a probability-based frameless sampling strategy. All respondents who entered the survey constituted the study sample. The target population was parents who were internet, social media, and institutional users. Via payment, Facebook promotes our survey to this specific audience. In terms of coverage, it is widely recognized that online surveys using only samples of internet users do not generalize to the general public. However, with more than 1.65 billion active users, Facebook is the largest online collection of socio-demographic information, constituting an effective way to get fast research responses. Moreover, Greece has one of the highest numbers of internet users (8.30 million internet users, 79% of the population, January 2020) and social media users (6.20 million, 56% of the population, January 2020) in the world [23]. In this study, the completion rate, which is the number of completed surveys compared to the number of respondents who entered the survey, was 90%.

The survey included 48 survey questions regarding the compliance of children with COVID-19 lockdown restrictions and the impacts of school closure on several educational, social, economic, and psychological parameters in children (Supplementary Materials: https://docs.google.com/forms/d/e/1FAIpQLSd3y1Lg2NbEr6xgGetUv_rBQ4P0C2FgF-W_KlPO848PA-_EFw/viewform). The questionnaire consisted of simple YES/NO questions, free questions and questions of the Likert scale of five points, such as “bad”, “moderate”, “good”, “very good”, and “excellent” or “not at all”, “little”, “moderate”, “enough”, and “very much”. The questionnaire was designed to be completed in less than 8 min. All participants responded to the e-survey after providing written consent. Participants were free to reject the survey at any time without prior justification. All study procedures involving human participants were conducted under the ethical standards of the institutional and/or national research committee and in accordance with the 2013 Helsinki Declaration and its later amendments or comparable ethical standards. Exclusion criteria included parents whose children did not attend primary education (4–12 years old) or those who did not reside in Greece. When exclusion criteria were met, the responses were marked as “Excluded from Analysis” and were not included in the exported dataset. Parents who did not have a personal Facebook account, had no web access, were illiterate, or could not use the technology, inevitably met the exclusion criteria.

### 2.2. Statistical Analyses

All categorical data were reported as percentages and differences in categorical data between groups were tested by two-tailed Pearson’s, chi-squared or Fisher’s exact test. A correction for continuity was applied. Numerical data were reported as mean and standard deviation. Differences in numerical data between groups were tested by two-tailed Student’s *t*-test, or Mann–Whitney test. Bivariate analyses of correlations of numerical data between groups were tested by Pearson’s correlation, r. *p*-Values smaller than 0.05 were considered significant. All statistical calculations were performed with IBM SPSS Statistics 20 software and Microsoft Excel.

## 3. Results

**Figure 1:**
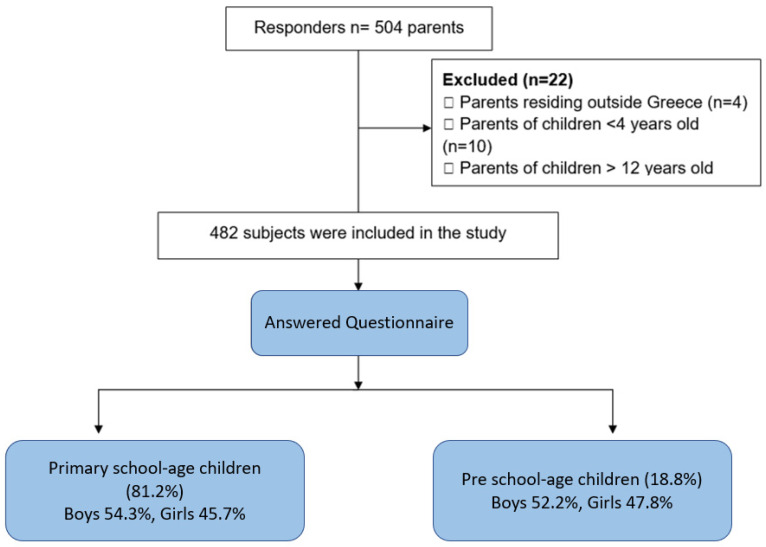
Flowchart of the study.

The workflow of our study is presented in Figure 1. Among 504 participants, 482 responses were deemed suitable for inclusion in the study after rigorous screening.

**Figure 2:**
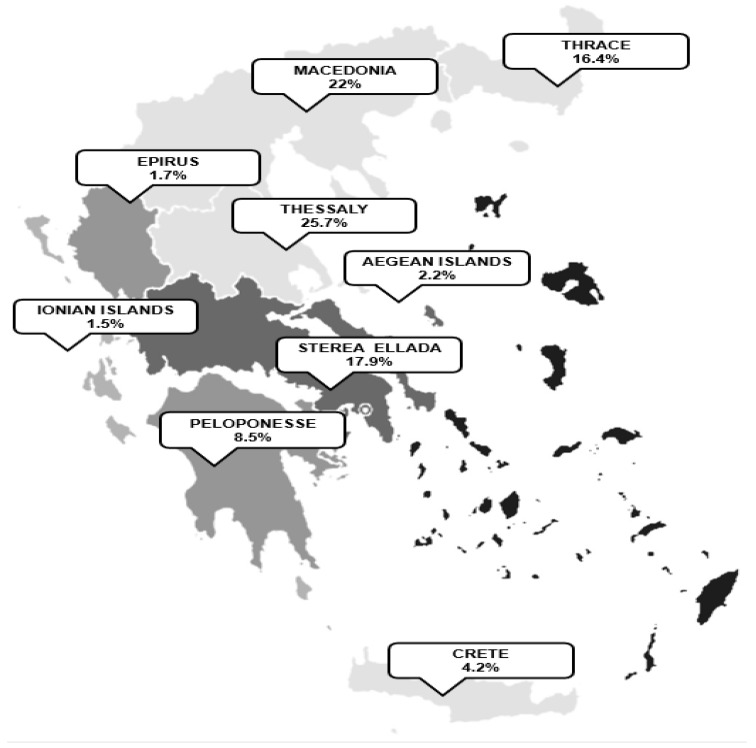
The distribution of respondents across Greece.

The distribution of respondents across Greece is visualized in Figure 2.

According to parents’ responses, the average age of the children was 8.1 ± 2.2 years. Seventy percent of the parents cared for at least two children. The average family sizes were 4 ± 1 people. Twenty-seven percent of the parents reported at least one family member suffered from a chronic disease. In seven percent of the families, there was a child with a chronic disease. Asthma was the most common disease among children, followed by diabetes, epilepsy, and polyallergy as shown in Table 1.

Forty-one (8.5%) of the respondents reported at least one confirmed COVID-19 case in their family, of these, 29.3% (*n* = 12) reported that their children got infected, one-third of these being asymptomatic.

Ninety-three percent of the parents reported that the schools complied with all requirements and obligations concerning COVID-19 prevention and hygiene measures. An overview of the measures in place can be found elsewhere [24]. In brief, mask usage in closed common areas was among the primary prevention measures. Other indicative interventions in place were regular ventilation, smaller activity groups (i.e., physical education, teaching, school canteen function, and school celebrations) and hygiene measures such as the school-based distribution of hand sanitizer.

**Figure 3:**
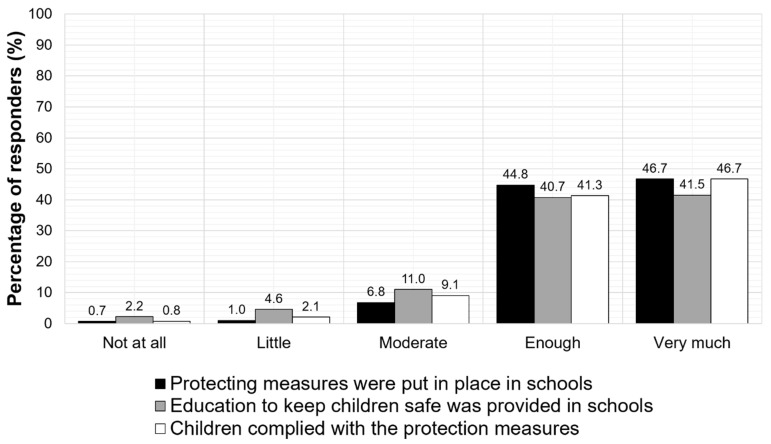
Parent-reported compliance of school environments and children with the required restriction and hygiene measures.

Eighty-four percent of the parents also supported that the teaching environments provided effective hygiene education for children and 85% of them documented that children had adopted the recommended hygiene measures, as shown in Figure 3.

Thirteen percent of the parents had not adopted the recommended hygiene measures. Interestingly, children adopted measures independently of their parents’ attitudes, as parents reported. Moreover, 39% of the respondents disagreed with the school closure as a protection measure against COVID-19 spread.

Only 11% of the parents claimed that the school was temporarily shut down before the general school closure. The reported average number of confirmed COVID-19 cases that led to school suspension was 3 ± 1 children. One-third (31%) of the respondents reported that some school classes, and not the whole school unit, were temporarily suspended to prevent the spread of the epidemic before the national decision of general school closure. Notably, in Greek cities overwhelmed with COVID-19 cases, there was a higher percentage of parent-related reports that a classmate of their children did not wear a mask into the classroom than in cities with lower COVID-19 incidence rates (32% vs. 22%, *p* = 0.002). Interestingly, 91% of participants supported that the presence of a health professional in the school environment could be protective against COVID-19.

A total of 90.7% of the respondents reported increased engagement of children in electronic media during the lockdown period, especially after the school closure. A total of 61.6% of the parents reported electronic overuse (playing online games, music listening, and movie watching) after the school closure, expressing their disappointment. Moreover, 48% of parents believed that school closures caused by COVID-19 could be even more damaging given that they led to a reduction in their children’s academic performance.

**Figure 4:**
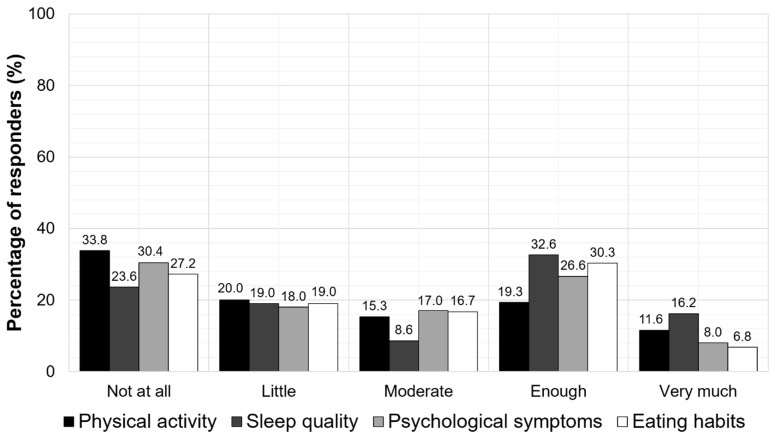
The parent-reported impacts of the school closures on physical activity, sleep quality, psychological symptoms, and eating habits of the children.

Furthermore, the school closure was found to negatively impact the children’s physical activity, psychological status, sleep quality, and eating habits. More specifically, almost 50% of the parents reported that their children had bad or moderate physical activity (53.8%), poor (bad or moderate) sleep quality (42.6%), psychological symptoms (48.4%), and unhealthy eating habits (46.2%). According to the parents, the mean sleep time of their children was 10.5 ± 1.2 h, and the time of awakening was 8.8 ± 1 h during the school closure. The parent-reported impacts of school closures on the physical activity, sleep quality, psychological symptoms, and eating habits of the children are presented in Figure 4.

**Figure 5:**
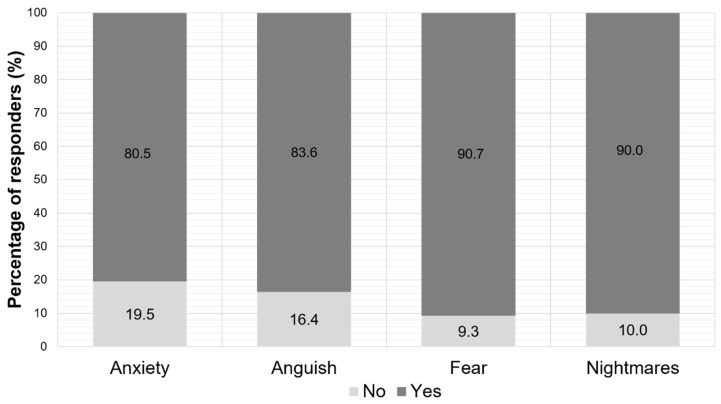
Parent-reported psychological symptoms of their children during the period of the school closure.

As far as the psychological symptoms are concerned, more than 80% of the parents claimed that their children had anxiety, anguish, fear, and nightmare disorders during school closure (Figure 5).

## 4. Discussion

This is the first Greek study investigating the parent-reported compliance of children aged from 4 to 12 years with COVID-19 lockdown restrictions and evaluating the impact of school closure on educational, social, economic, and psychological outcomes in children. We found that the school units were well-informed and complied with the protective hygiene measures against COVID-19. The pupils quickly adopted the protection measures, even those whose parents did not believe in the effectiveness of mask-wearing. Almost half of all families reported that the quarantine period negatively impacted physical activity, quality of sleep, psychological status, eating habits, and the primary school children’s academic performance.

Our research detected only a small proportion of infected and symptomatic children. It has been suggested that children are less likely to spread the SARS-CoV-2, and fatal cases in children are rare [9,25,26]. The minor role that children played in the virus transmission brings up the question of the impact of school closure in reducing COVID-19 spread. The WHO report summarizes that the children’s infection rate ranged between 3 and 10% in China, while no evidence of transmission from infected children to adults was found [25]. Furthermore, according to the German Society for Pediatrics and Adolescent Medicine, 81% of children are infected by their parents [25]. Moreover, in overwhelmed areas with large numbers of infected adults, there were solely isolated pediatric cases [26]. In a systematic literature review by the Norwegian Institute of Public Health, the authors concluded that children appeared to be less susceptible to symptomatic SARS-CoV-2 infection than adults and the role of children in viral transmission was not significant [9].

Proper handwashing promotes wellness in childcare [27,28]. Rabie et al. argued that hand washing could break the transmission cycle and reduce the risk of COVID-19 [29]. Although hand washing is a cheap and widely available prevention measure to reduce infection risk, it is not easily implemented by children [30,31]. Similarly, the use of a mask by first graders has difficulties. Apart from the size of the mask that must fit perfectly on children’s faces, compliance remains an issue [32]. The present study showed that most school environments developed specific COVID-19 prevention messages for students with an emphasis on proper handwashing procedures and facemask wearing, resulting in children’s compliance with protective hygiene measures.

The majority of parents accepted that the school closure and mask wearing are proactive measures to restrict the virus spread, and only a small proportion of respondents believed that they did not need to wear a mask. Although children are used to copying parental behavior [33], we noted that the pupils adopted the protection measures even if parents did not believe in the effectiveness of mask-wearing. This finding highlights the major role that schools play by developing an appropriate hygiene education campaign aimed at children.

According to the majority of parents (91%), the presence of a health professional in a school environment can act protectively during a pandemic. The role of health professionals in schools was revisited in Greece, both in the sense of heightened vigilance for respiratory symptoms, and as an on-site liaison in case of an outbreak [24]. This point-of-care concept is likely to have contributed towards the positive view captured by our study.

We also found that the lockdown period, especially after the school closure, harmed the physical activity, psychological status, sleep quality, eating habits, and academic performance of the primary school-age children. Our results are in line with previous studies that documented that children have reduced physical activity given that they are not accessing school activities due to the threat of the pandemic [17]. Social distancing and isolation impact children’s psychology [17]. Most children seemed to be under-stimulated due to the school closure [17,34]. The web appears to be the only means of socialization and distance learning during the pandemic [17,34]. Accessing vast amounts of data (messaging, photos, audio, live streaming, and meeting rooms) leads to over-occupancy with the web [34]. E-learning cannot replace in-person teaching and reduces children’s academic performance, especially in first graders where hand–eye coordination is needed for writing [10,11].

Almost half of the respondents reported children’s impaired psychological status. Moreover, more than 80% of the parents claimed that their children had anxiety, anguish, fear, and poor sleep quality with nightmare disorders during school closure, although the average sleep duration reported by parents was not negatively impacted. The most prevalently reported adverse psychological effect of school closure was fear, closely followed by nightmares. Isolation carries a multitude of psychological burdens and various neuropsychiatric manifestations (anxiety, distress, and fear) [13]. Isolation under stressful conditions also impacts the quality of sleep. Sleep plays an essential role in children’s development and is essential for their physical and mental health [17]. Other studies estimated that 25% of all children experienced at least one sleep-related disorder during the quarantine [35]. It has been reported that poor sleep quality leads to daytime drowsiness, headaches, behavioral problems and affects health and life quality and, subsequently, poor academic performance [17,36].

In addition, almost half of the respondents reported unhealthy eating habits in children. Despite the short-term nature of the current study to make conclusions, this finding is consistent with that of Androutsos et al., who reported an increase of body weight by 35% of children/adolescents during the first COVID-19 lockdown in Greece associated with increased consumption of breakfast, salty snacks, and total snacks, and with decreased physical activity [37]. According to parents, children adopted unhealthy eating habits while staying at home. Specifically, the quarantine period enhanced stress levels leading to the overconsumption of foods rich in sugar and other simple carbohydrates [18,19]. Although eating sugar reduces stress and serotonin secretion, it is also associated with an increased risk of obesity [19,38,39]. In addition to economic and social instability, it has been reported that home isolation measures can affect the food supply chain and create a state of food insecurity [19,38]. Studies supported that during the pandemic, malnutrition and obesity can increase due to limited access to healthy food preparation materials, limited gastronomic variability, and a sedentary lifestyle [19,38,39]. Consumption of processed foods rich in calories, saturated fats, sugars and refined carbohydrates, especially in children, can help increase the prevalence of obesity in the COVID-19 era [39]. An association between sleep disorders and obesity has also been documented [40,41]. The increased secretion of pro-inflammatory cytokines by the surplus visceral fat could contribute to an altered sleep–wakefulness rate [40,41].

Our study’s findings should be interpreted within the framework of its limitations. As such, our study reports on effects evident after two weeks of lockdown. In this context, our findings refer to its immediate, rather than long-term consequences. Respondent bias could be present in our sample, manifesting as an increased response propensity in parents whose children suffer from chronic disease. Moreover, the “moderate” option, could be construed as a positive or negative and respondents could have interpreted this differently. Selection bias could similarly affect our findings, since dissemination of the questionnaire itself and its discoverability was inescapably dictated by the means of exposure to the public. Certain populations were difficult to reach as they were less likely to have internet access, a personal Facebook account, or to respond to online questionnaires, which are associated with low response rates. Finally, the lack of a trained interviewer to clarify and probe could lead to less reliable data.

Although the findings should be interpreted with caution, this study has several strengths. A key strength of this study was that we used an identification verification tool provided by Facebook that demands ID verification for questionnaires in order to prevent one person from submitting multiple responses.

## 5. Conclusions

The present study highlights the major role that schools played in developing an appropriate hygiene education campaign aimed at children resulting in their compliance with protective hygiene measures, regardless of their parents’ beliefs, in Greece. On the other hand, almost half of all families reported that the quarantine period negatively impacted physical activity, quality of sleep, psychological status, eating habits, and children’s academic performance. Our study highlights the need for long-term monitoring of these aforementioned indices, and the development of COVID-19 mitigation measures that carefully incorporate effectiveness and societal impact.

## Figures and Tables

**Table 1:** Parent-reported health problems of pre-primary and primary school-age children among families with at least one child with a chronic disease.

Pediatric Diseases	Percentages
Asthma	29.6%
Diabetes	21.4%
Epilepsy	15.8%
Polyallergy/multiple chemical sensitivity	15.2%
Hereditary Thrombophilia	6.7%
Obesity	4.6%
Sickle cell anemia	3.3%
Pediatric multiple sclerosis	1.4%
Fabry disease	1.0%

Note: The percentages are expressed as a proportion of the 7% who indicated at least one comorbidity.

## Data Availability

The data presented in this study are available on request from the corresponding author. The data are not publicly available due to privacy.

## Supplementary Materials

The full questionnaire has been provided as supplementary material can be found on the following link (Greek) https://docs.google.com/forms/d/e/1FAIpQLSd3y1Lg2NbEr6xgGetUv_rBQ4P0C2FgF-W_KlPO848PA-_EFw/viewform.
